# Loss of Munc18-1 long splice variant in GABAergic terminals is associated with cognitive decline and increased risk of dementia in a community sample

**DOI:** 10.1186/s13024-015-0061-4

**Published:** 2015-12-02

**Authors:** Alfredo Ramos-Miguel, Christa Hercher, Clare L. Beasley, Alasdair M. Barr, Thomas A. Bayer, Peter Falkai, Sue E. Leurgans, Julie A. Schneider, David A. Bennett, William G. Honer

**Affiliations:** Child and Family Research Institute, 938 West 28th Avenue, Vancouver, BC V5Z 4H4 Canada; Department of Psychiatry, University of British Columbia, 2255 Wesbrook Mall, Vancouver, BC V6T 2A1 Canada; Department of Anesthesiology, Pharmacology and Therapeutics, University of British Columbia, 2176 Health Sciences Mall, Vancouver, BC V6T 1Z3 Canada; Department of Psychiatry, University Medicine Goettingen, von-Siebold-Strasse 5, D-37075 Goettingen, Germany; Department of Psychiatry and Psychotherapy, Ludwig-Maximilians-University Munich, Nussbaumstrasse 7, D-80336 Munich, Germany; Rush Alzheimer’s Disease Center, Rush University Medical Center, 600S. Paulina Street, IL 60612 Chicago, USA

**Keywords:** Syntaxin-binding protein, SNARE, Protein-protein interactions, VGAT, VGLUT1, Human postmortem brain, Aging study, Mild cognitive impairment, Synaptic pathology, Alzheimer’s disease

## Abstract

**Background:**

Presynaptic terminals contribute to cognitive reserve, balancing the effects of age-related pathologies on cognitive function in the elderly. The presynaptic protein Munc18-1, alternatively spliced into long (M18L) or short (M18S) isoforms, is a critical modulator of neurotransmission. While subtle alterations in Munc18-1 have been shown to cause severe neuropsychiatric disorders with cognitive impairment, little information is known regarding the specific roles of Munc18-1 splice variants. We first investigated functional and anatomical features evidencing the divergent roles of M18L and M18S, and then evaluated their contribution to the full range of age-related cognitive impairment in the dorsolateral prefrontal cortex of a large sample of participants from a community-based aging study, including subjects with no-(NCI, *n* = 90), or mild-(MCI, *n* = 86) cognitive impairment, or with clinical dementia (*n* = 132). Finally, we used APP23 mutant mice to study the association between M18L/S and the time-dependent accumulation of common Alzheimer’s disease pathology.

**Results:**

Using isoform-specific antibodies, M18L was localized to the synaptosomal fraction, with a distribution matching lipid raft microdomains. M18S was found widely across cytosolic and synaptosomal compartments. Immunocytochemical studies identified M18L in perisomatic, GABAergic terminals, while M18S was broadly distributed in GABAergic and glutamatergic terminals. Using regression models taking into account multiple age-related pathologies, age, education and sex, global cognitive function was associated with the level of M18L (*p =* 0.006) but not M18S (*p =* 0.88). Mean M18L in dementia cases was 51 % lower than in NCI cases (*p <* 0.001), and each unit of M18L was associated with a lower likelihood of dementia (odds ratio = 0.68, 95 % confidence interval = 0.50–0.90, *p =* 0.008). In contrast, M18S balanced across clinical and pathologically diagnosed groups. M18L loss may not be caused by age-related amyloid pathology, since APP23 mice (12- and 22-months of age) had unchanged cortical levels of M18L/S compared with wild-type animals.

**Conclusions:**

M18L was localized to presynaptic inhibitory terminals, and was associated with cognitive function and protection from dementia in an elderly, community-based cohort. Lower M18L in inhibitory presynaptic terminals may be an early, independent contributor to cognitive decline.

**Electronic supplementary material:**

The online version of this article (doi:10.1186/s13024-015-0061-4) contains supplementary material, which is available to authorized users.

## Background

Common age-related neuropathologies, including Alzheimer’s disease pathology (i.e. neuritic plaques and neurofibrillary tangles), cerebral infarcts, and Lewy bodies, accumulate in the human brain across the lifetime. The susceptibility of cognitive function to decline as growing amounts of neuropathology develop in the forebrain varies greatly between individuals, raising the concept of cognitive reserve [1–3]. Large community-based studies have been designed, at least in part, to investigate the biological substrates of cognitive reserve [4–7]. The initial model that people with mentally enriched lives may exhibit greater resistance against brain-damaging diseases [2] is not yet supported with clear neurochemical correlates. Synaptic pathology is common in aging, being first reported as a deficit of presynaptic markers [8–11]. In some studies, synaptic pathology showed stronger association with cognitive decline than other common age-related pathologies [12], suggesting that intact, functional synapses may contribute to cognitive reserve.

In this context, we previously reported that better cognitive performance, a reduced odds of dementia, and slower rate of cognitive decline over multiple years prior to death were associated with greater densities of specific presynaptic proteins, and enhanced presynaptic protein-protein interactions (PPIs) [13, 14]. Interestingly, these PPIs involved the associations between syntaxin-1, synaptosomal-associated protein-25 (SNAP-25), and vesicle-associated membrane protein (VAMP, or synaptobrevin), which together assemble the machinery facilitating vesicle trafficking and neurotransmitter release, known as the soluble *N*-ethylmaleimide-sensitive factor attachment protein receptor (SNARE) [15]. However, other frequent targets of synaptic pathology, such as synaptophysin, were not associated with cognitive function or Alzheimer’s disease pathology in the same community-based cohort study, which included subjects with the full range of cognition from normality, to mild cognitive impairment, to dementia. Of note, many of the studies related to synaptic pathology were based on subpopulations of hospital- or clinic-based patients displaying moderate-to-severe degrees of dementia, not necessarily representative of the spectrum of cognitive decline in aging [16]. Previous analyses in mixed postmortem brain series found biphasic alterations of brain presynaptic markers (including synaptophysin) across age-related cognitive decline and/or Alzheimer’s disease progression, with upregulated densities at intermediate stages and reduced levels in more severe cases [17–19]. Together, these observations suggest that an initial failure of presynaptic terminals to maintain synaptic function (reflected by SNARE disruption) might be compensated by upregulating the expression of certain presynaptic genes; further accumulation of neuropathologies may result in the observed synaptic degeneration at relatively more severe stages. The mechanisms underlying initial SNARE and presynaptic dysfunction could be directly linked to age-related cognitive impairment and progression to dementia. Consistent with these observations, murine models with impaired SNARE PPIs undergo related behavioral and physiological neurodegenerative syndromes [20, 21].

Although the biochemical and physiological nature of neurosecretion is well established [22–24], the association between SNARE dysregulation and the pathophysiology of aging-related diseases is unclear. Among the regulators potentially involved in SNARE dysfunction, mammal *unc*-18-1 (Munc18-1 or n-Sec1; herein M18) is a strong candidate. First identified by random mutagenesis screening for *unc*oordinated phenotypes in the worm *Caenorhabditis elegans*, M18 caught the attention of many research teams when described as a ‘syntaxin-binding protein’ (coining the M18 coding gene as *STXBP1*) twenty years later [25–27]. While still controversial, M18 is believed to have multiple activities during SNARE-regulated exocytosis, which may include trafficking and chaperoning syntaxin-1, transition from *cis*-to-*trans* SNARE conformations, and/or docking the presynaptic vesicle [23, 28, 29]. The observation that *STXBP1* null mice lack neocortical synaptic activity [30] highlights the irreplaceable role of M18 for neurosecretion. Interestingly, M18 knockout mice also exhibit massive neuronal apoptosis and widespread neurodegeneration, and consequently do not survive after birth [30].

**Fig. 1:**
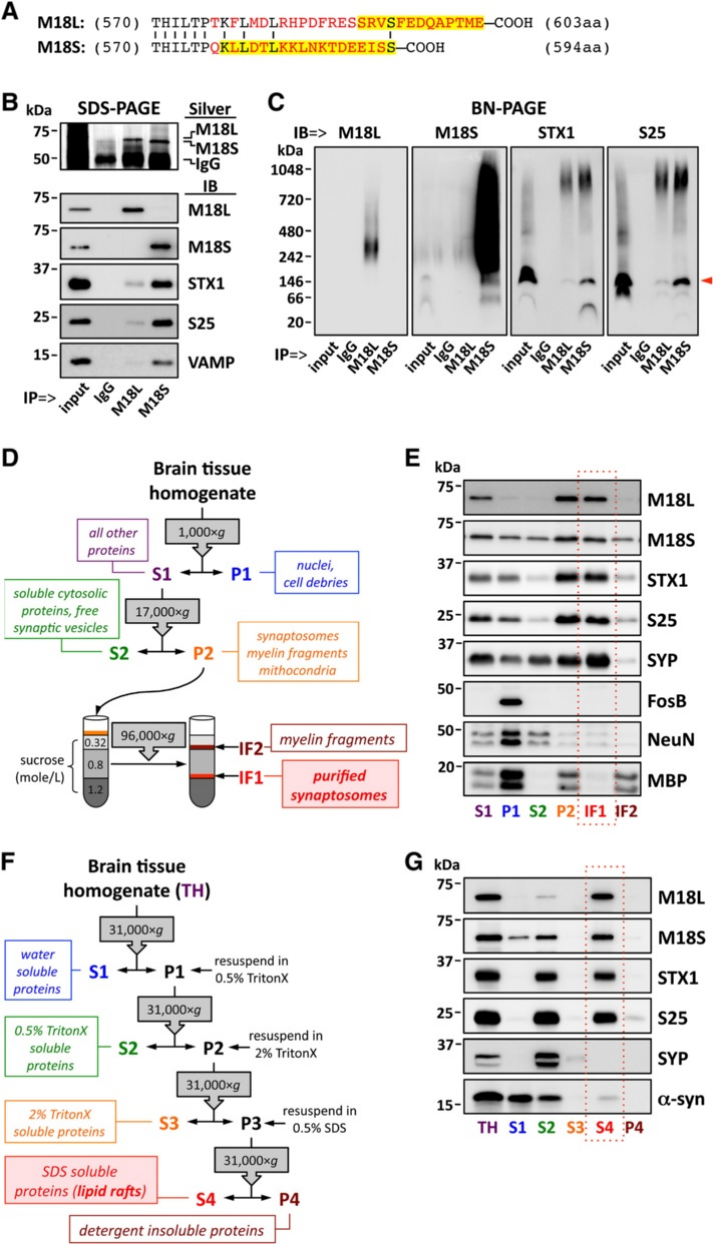
Biochemical characterization of M18 splice variants. **a** Alignment of M18L/S C-terminal amino acid (aa) sequences. Mismatching residues are in red. Amino acids highlighted (in yellow) represent the immunogenic sequences used for production of variant-specific antibodies. Omitted M18L/S N-terminal sequences are 100 % identical. **b**–**c** Immunoprecipitation (IP) of M18L/S with variant specific antibodies (and anti-mouse IgG as a negative control) using human brain homogenates. NCI control subjects (*n* = 3) were tested with similar results. IP products, along with input samples and negative controls (IgG), were resolved by (**b**) standard SDS- or (**c**) BN-PAGE, followed by either silver staining (Silver) or immunoblotting (IB) with specific antibodies against M18 variants, syntaxin-1 (STX1), SNAP-25 (S25), or VAMP. **c** M18S antibody recognized two bands at ~150 and ~70 kDa, putatively corresponding to a SNARE-M18 heterotetramer (pointed with a red arrowhead) and the monomeric form, respectively. **d, f** Schematic illustration depicting the sequential extraction of (**d**) synaptosomes and (**f**) lipid rafts from human cortical homogenates. *g*, relative centrifugal forces; IF, interface; P, pellet; S, supernatant. **e** Equivolumetric amounts of fractions obtained in (**d**) were resolved by SDS-PAGE and immunoblotted using antibodies targeting M18L/S, STX1, S25, VAMP, and markers for synaptic vesicles (synaptophysin [SYP]), nuclei (FosB), nuclei + cytosol (NeuN), and myelin fragments (myelin basic protein [MBP]). Note that MBP strongly labels P1, as heavy myelin fragments precipitate along with cell debris. **g** Equivolumetric amounts of fractions obtained in (**f**) were resolved by SDS-PAGE and immunoblotted using the antibodies above. α-Synuclein (α-syn) antibody was additionally used, showing similar distribution across fractions as previously reported [66]. **e**, **g** IF1 and S4 fractions were framed in a red, dashed box to highlight synaptosome and lipid raft-enriched proteins, respectively. **b**–**g** Masses (in kDa) of prestained markers are indicated on the left side of immunoblots

In mammals, the *STXBP1* gene is alternatively spliced to yield either a long (M18L) or a short (M18S) variant [31, 32], also called isoforms *a* and *b*, respectively. Processing of the final exon in the *STXBP1* primary transcript may include (M18L) or skip (M18S) a sequence of 110 bp containing a stop codon [32], resulting in two different C-terminal amino acid sequences for M18L/S (see Fig. 1a). To our knowledge, specific regulatory mechanisms of *STXBP1* gene splicing have not been described. Although intensive research focuses on M18 activities in regulated exocytosis, the potentially divergent roles of the M18 variants are relatively neglected, assuming no substantial differences between them [32]. However, recent observations indicate that M18 variants may not have overlapping functions. Transgenic mice expressing a fluorophore-tagged, functional M18S were generated using a replacement strategy that erased the M18L splice variant [33, 34]. These animals show severe physical and behavioral deficits, and die prematurely a few weeks after birth. Conversely, mice overexpressing M18L in both glutamatergic and GABAergic neurons display aspects of a schizophrenia-like phenotype [35], compatible with alterations observed in human postmortem studies [36–38]. At a cellular level, transfection of either M18L or M18S to excitatory neuronal cell cultures from the hippocampus of M18 knockouts succeeded to a similar extent in rescuing basal synaptic activity [39]. However, M18S appeared to support high frequency stimulation more efficiently than the long variant, suggesting different roles in short-term plasticity. Altogether, these findings indicate that the functions of M18 splice variants may not be interchangeable, and the multiplicity of activities attributed to M18 may represent a composite of M18L + S.

It has been reported that *de novo* mutations affecting *STXBP1* gene can cause Ohtahara syndrome in humans [40–45], a devastating neurological disease characterized by early onset of epileptic seizures and a profound intellectual disability. Most *STXBP1* mutations associated with Ohtahara syndrome impair M18–syntaxin-1 interaction (and thereby proper SNARE assembly), compromising cellular availability of functional M18 (haploinsufficiency) [41, 43]. Conversely, other mutations are predicted to confer aberrant alternative splicing of M18 RNA [44]. In addition, some *STXBP1 de novo* mutations were associated with similar mental retardation without epileptic seizures [42].

In the context of aging, two previous studies addressed alterations in M18 protein levels in Alzheimer’s disease postmortem brain. In the first, M18 immunodensity was lower in two cortical areas in *n =* 32 Alzheimer’s disease cases, although after synaptophysin normalization, the authors concluded that productive synapses in Alzheimer’s disease brains might be enriched in M18 [46]. A later proteomic study [47] found increased M18 levels in cell membrane extractions from cortical samples of *n =* 5 Alzheimer’s disease cases. These two studies did not examine the correlation of M18 with cognitive outcomes, or with the likelihood of dementia; nor were potential differences in M18 splice variants assessed. In addition, the small sample sizes limited the possibility of investigating possible effects of stage of illness, or potential confounders.

Altogether, clinical and preclinical data demonstrated that M18 is essential for neurosecretion, subtle alterations have a dramatic impact on normal synaptic function and cognition in humans and rodents, and could represent an early sign of synaptic pathology and cognitive impairment. For the present study we hypothesized that cortical downregulation of M18 long and/or short (M18L/S) splice variants could contribute to poorer cognitive performance and increased likelihood of dementia in old age. Due to the scarce data on specific functions of M18 isoforms, we first performed functional and anatomical characterization experiments that evidenced the complementary, rather than overlapping roles of M18L and M18S. Using tissue samples from the community-based Memory and Aging Project (MAP) [7], cortical expression of M18 variants was quantified in a large sample of MAP participants with and without clinical dementia, representing the range of cognitive impairment in an elderly, aging population. The potential effects of Alzheimer’s disease pathology on M18 levels were further modeled using APP23 transgenic mice, which overexpress a pathogenic variant of the human amyloid precursor protein (APP) [48].

## Results

### Biochemical and anatomical evidence indicates different roles of M18 splice variants

We first used immunoprecipitation (IP) followed by either sodium dodecyl sulfate (SDS) or blue native (BN) polyacrylamide gel electrophoresis (PAGE) to investigate functional binding affinities of M18 isoforms for the SNARE complex. As expected from their target immunogenic sequences (Fig. 1a), and also from previous data [36], variant-specific antibodies selectively immunoprecipitated M18L or M18S without cross-reacting, confirmed by immunoblot analyses (Fig. 1b). Surprisingly, anti-M18S IP products were 7–12 fold enriched in SNARE proteins (i.e. syntaxin-1, SNAP-25 and VAMP), compared to those of anti-M18L (Fig. 1b). This was not due to a lower affinity of the antibody for M18L, since comparable amounts of both isoforms were observed in silver stained gels (Fig. 1b). Rather, M18L displayed (under current assay conditions) a poorer ability to bind syntaxin-1. Accordingly, M18S formed a stable ~150-kDa complex with SNARE proteins, which was barely detected in M18L co-IP products, as revealed by BN-PAGE (Fig. 1c).

Substantial differences in the subcellular localization of M18 variants were also observed (Fig. 1d–g). While M18L was apparently restricted to the synaptosomal fraction (IF1), M18S showed the expected wide distribution across cytosolic (S2) and synaptosomal compartments (Fig. 1e). Furthermore, M18L was almost exclusively found within the Triton X-100 (TritonX)-insoluble, SDS-soluble protein fraction (S4), matching lipid raft microdomains, while M18S was present in water- and detergent-soluble subcellular compartments (Fig. 1g).

**Fig. 2:**
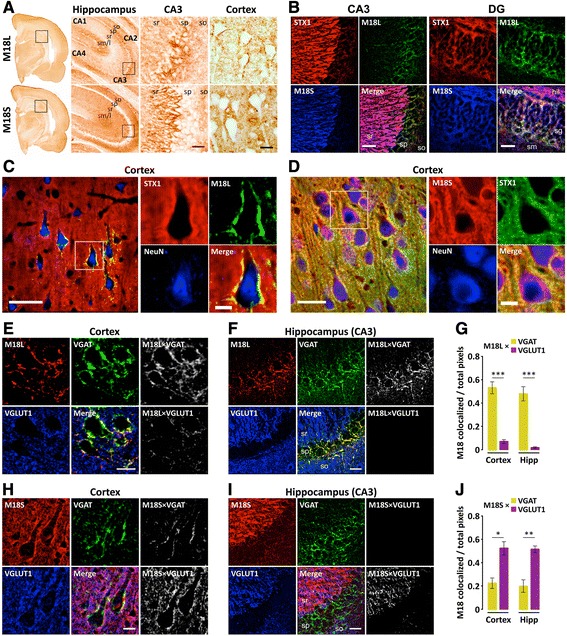
Immunohistochemical characterization of M18 splice variants in rat brain. (**a**) Photomicrographs showing M18L (*upper panels*) and M18S (bottom panels) immunostaining with variant specific antibodies at various magnifications. Hippocampus and CA3 are magnified captions from the framed areas at immediate left-side images. (**b**–**j**) Confocal images and analyses from triple co-immunolabeled sections with the antibodies indicated at the top-left corners. Colors were arbitrarily assigned to maximize overlap visualization. (**e**–**j**) Rat brain sections were co-immunolabeled with antibodies against vesicular GABA (VGAT) and glutamate (VGLUT1) transporters, along with (**e**–**f**) anti-M18L or (**h**–**i**) anti-M18S, and representative images from (**e** and **h**) cortical and (**f** and **i**) hippocampal CA3 are shown. In every 6-panel composite: left and middle panels correspond to single-labeled or merged-channel images; right panels are ImageJ-generated bitmaps resulting from colocalization analyses, in which pixels mirror the intensity of colocalization (in white) between VGAT (top) or VGLUT1 (bottom) and the corresponding M18 splice variant. (**g** and **j**) Quantitative colocalization analyses of (**G**) M18L or (**J**) M18S with each of the vesicular transporters (VGAT and VGLUT1) Bars represent mean ± standard error of *n =* 4 rats. *p < 0.05, **p < 0.01, and ***p < 0.001 (paired *t*-test). Abbreviations: DG, dentate gyrus; hil, hilus; Hipp, hippocampus sg; stratum granulosum (i.e. granule cell layer); sm/l, stratum moleculare/lacunosum; so, stratum oriens; sp, stratum pyramidale (i.e. pyralmidal cell layer); sr, stratum radiatum (in CA3 also contains stratum lucidum. Scale bars: **a**, 200 (CA3) or 20 (cortex) μm; **b**, 30 μm; **c**, 50 (*left*) or 10 (*right*) μm; **d**, 30 (left) or 10 (right) μm; **e** and **h**, 10 μm; **f** and **i**, 25 μm

Immunohistochemical analyses of rat brain revealed a differential distribution of M18 variants, particularly in the hippocampus (Fig. 2a). M18L was highly abundant in the pyramidal cell layer of the Ammon's horn (CA) regions, as well as in the dentate gyrus granule cell layer. In marked contrast, M18S showed greatest immunostaining in pyramidal cell-flanking strata (with particularly strong labeling of the mossy fibers), but was barely present within the pyramidal layer. In cortical regions, M18L accumulated in neuronal perisomatic areas, whereas M18S showed the punctate staining of neuropil typically observed for presynaptic proteins (Fig. 2a). Despite their apparently distinct patterns of distribution (best characterized in CA3), confocal imaging showed some degree of overlap between splice variants in areas such as the granule cell layer (Fig. 2b). Of note, colocalization of M18 isoforms also overlapped with syntaxin-1. Similar results were observed in human hippocampus (Additional file 1: Figure S1A). In NeuN co-stained sections, preferential perisomatic accumulation of M18L was highlighted, and additional evidence of the cytosolic localization of M18S (but not M18L or syntaxin-1) obtained (Fig. 2c–d).

**Fig. 3:**
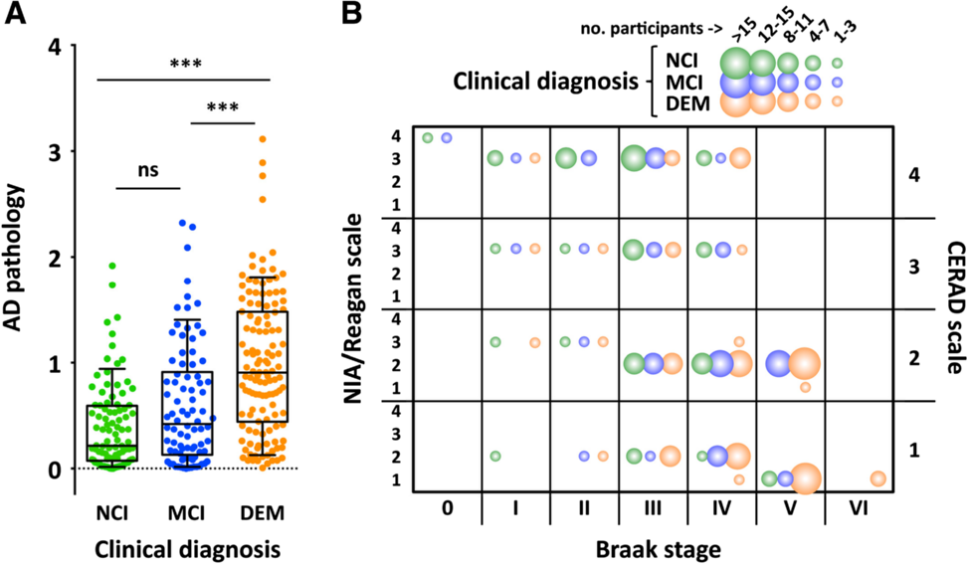
Burden of Alzheimer’s disease pathology in MAP participants and relation to clinical diagnoses. **a** Composite Alzheimer’s disease (AD) pathology values were estimated for each participant and plotted by clinical diagnosis criteria into no- (NCI, *n =* 90), or mild-cognitive impairment (MCI, *n =* 86), or dementia (DEM, *n =* 132). Whiskers represent 10th and 90th percentiles and boxes enclose interquartile ranges crossed by the median of Alzheimer’s disease pathology scores within groups. As expected, Kruskal-Wallis test detected differences on the accumulated Alzheimer’s disease pathology across clinical diagnoses (KW-statistic = 57.0, *p <* 0.001; Mean rank differences: NCI *vs* MCI = 30.7; NCI *vs* DEM = 88.7; MCI *vs* DEM = 58.0). ns, not significant, ***p < 0.001, Kruskal-Wallis followed by Dunn’s multiple comparison test. **b** Bubble plot illustrating the distribution of MAP participants across clinically diagnosed (NCI/MCI/DEM) and neuropathologically graded [by NIA/Reagan, CERAD (Consortium to Establish a Registry for Alzheimer’s Disease), and Braak scales] groups

Triple co-immunolabeling with antibodies against M18L, vesicle GABA (VGAT) and glutamate (VGLUT1) transporters showed a high degree of M18L localization within the inhibitory (VGAT-positive) synapses, with little (or possibly no) presence at excitatory (VGLUT1-positive) terminals, in both cortex and hippocampus (Fig. 2e–g). In contrast, overlap of M18S was greater with VGLUT1 than with VGAT (Fig. 3h–j), although this could be explained by the relative abundance of glutamatergic over GABAergic terminals. This difference was most remarkable in CA3, where a complete segregation of M18L and M18S with respectively, VGAT or VGLUT1 positive terminals was observed (Fig. 2f, i). In human hippocampus, similar distributions were seen (Additional file 1: Figure S1B–C).

### Characteristics of MAP participants and potential confounds

Demographic, cognitive and pathological variables of MAP participants are summarized in Table 1. No correlation was detected between M18 splice variants and potential confounders, including age at death, postmortem interval, years of education and brain weight. Similarly, M18L/S cortical immunodensity did not vary across sex, race, or ApoE genotype. Furthermore, tobacco and/or alcohol users did not present different M18L/S levels from those without these histories.

**Table 1:** Demographic, cognitive and pathological characteristics^*a*^ of MAP participants included in the present study

Variable	All participants (*n* = 308)	NCI (*n* = 90)	MCI (*n* = 86)	Dementia (*n* = 132)
*Demographic*				
Female, no. (%)	194 (63 %)	64 (71 %)	48 (56 %)	82 (62 %)
Age at death, years	88.8 ± 6.0	86.8 ± 6.3	88.1 ± 6.4	90.7 ± 5.0
Education, years	14.4 ± 2.8	14.0 ± 2.4	14.7 ± 2.5	14.5 ± 3.2
Race, no. W:AA:NA	300:7:1	86:4:0	84:1:1	130:2:0
*APOE* ε4 allele, no. (%)	84 (27 %)	15 (17 %)	22 (26 %)	47 (36 %)
PMI, hours	7.2 ± 4.8	6.9 ± 4.4	8.1 ± 5.5	6.7 ± 4.6
*Cognitive function proximate to death*				
Global cognition score	−0.85 ± 1.09	0.16 ± 0.41	−0.43 ± 0.45	−1.83 ± 0.88
Episodic memory	−0.84 ± 1.19	0.34 ± 0.48	−0.55 ± 0.70	−1.84 ± 0.90
Semantic memory	−0.70 ± 1.23	0.12 ± 0.56	−0.19 ± 0.53	−1.61 ± 1.31
Working memory	−0.66 ± 1.14	0.16 ± 0.77	−0.29 ± 0.79	−1.50 ± 1.01
Perceptual speed	−1.13 ± 1.13	−0.28 ± 0.85	−0.70 ± 0.88	−2.02 ± 0.77
Visuospatial ability	−0.63 ± 1.23	0.12 ± 0.62	−0.16 ± 0.78	−1.49 ± 1.29
MMSE	21.3 ± 8.8	28.1 ± 1.8	25.7 ± 3.8	13.6 ± 8.2
*Pathological*				
NIA/Reagan scale, no. in 1:2:3:4^*b*^	49:135:120:4	4:25:59:2	4:44:36:2	41:66:25:0
CERAD scale, no. in 1:2:3:4^*c*^	95:99:40:74	13:21:19:37	19:31:13:23	63:47:8:14
Braak stage, no. in 0:I:II:III:	4:20:33:91:	2:14:16:33:	2:2:14:28:	0:4:3:30:
IV:V:VI	88: 67:5	21:4:0	30:10:0	37:53:5
Global AD pathology	0.69 ± 0.62	0.38 ± 0.39	0.60 ± 0.57	0.97 ± 0.66
Macroinfarcts, no. (%)	108 (35 %)	20 (22 %)	29 (34 %)	59 (45 %)
Microinfarcts, no. (%)	71 (23 %)	20 (22 %)	14 (16 %)	37 (28 %)
Lewy body disease, no. (%)	27 (9 %)	2 (2 %)	4 (5 %)	21 (16 %)
Hippocampal sclerosis, no. (%)	26 (8 %)	1 (1 %)	5 (6 %)	20 (15 %)

Abbreviations: *AA* Afro-American, *AD*, Alzheimer’s disease, *BL* baseline, *CERAD* Consortium to establish a registry for AD, *MCI* mild cognitive impairment, *MMSE* mini mental state examination, *NA* Native-American, *NCI* no cognitive impairment, *NIA* National Institute on Aging, *no* number of subjects, *PMI* postmortem interval, *SD* standard deviation, *W* White

^*a*^Values are mean ± SD unless noted otherwise

^*b*^NIA/Reagan scale: high (1), intermediate (2), low (3), or no (4) likelihood of AD, according to neuritic plaques and tangles

^*c*^CERAD scale: definite (1), possible (2), probable (3), or no (4) AD, according to neuritic plaques

Similar numbers of clinically diagnosed MAP participants with no (NCI) or mild (MCI) cognitive impairment, and dementia were observed (Table 1). As expected, common Alzheimer’s disease pathology was largely abundant in dementia cases, compared to NCI and MCI participants (Fig. 3a), although some degree of variability was present in all clinically diagnosed groups, as documented previously in larger epidemiologic studies [5, 7]. Similarly, clinical diagnoses showed substantial, but not complete, overlap with CERAD and Braak rating scales (Fig. 3b).

### Association of cortical M18L with cognitive function and dementia

We investigated possible alterations in the amounts of M18 splice variants, and their potential association with cognitive function, in samples from the dorsolateral prefrontal cortex (DLPFC) of MAP participants (*n =* 308). Initial inspection of quantitative immunoblotting datasets (normalized by β-actin content) revealed large variability in M18L immunodensities among MAP participants (interquartile range = 7–185 %), compared to that of M18S (86–122 %) or β-actin (85–117 %). The immunodensities of the M18 variants showed a non-linear association (Additional file 1: Figure S2A, Spearman rho = 0.53), best fitted by a semi-log curve (*r =* 0.56, *p <* 0.001). Due to the large number of values proximate to zero (see Additional file 1: Figure S2), and consequent skewness (skew = 1.712), the distribution of M18L values did not pass the Kolmogorov-Smirnov normality test. To construct linear models, we log-transformed M18L values, and then standardized (by subtracting the mean and dividing by the standard deviation) both variant datasets. Data transformations symmetrized the distribution of M18L values and rendered linear associations between the variants (*r =* 0.51, *p <* 0.001).

**Fig. 4:**
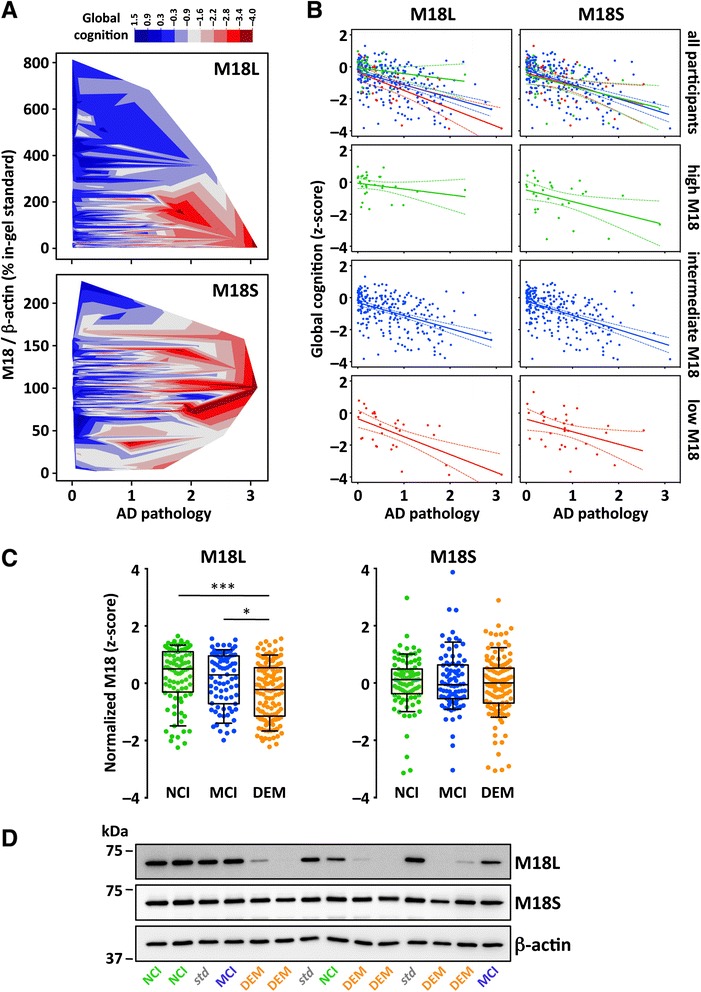
Associations of M18 splice variants with Alzheimer’s disease pathology, global cognition and clinical dementia. **a** Contour plots illustrating MAP participants’ (*n =* 308) global cognition (z-score) as a function of M18L or M18S DLPFC immunodensities, and Alzheimer’s disease (AD) pathology. Only AD pathology × M18L (*r =−*0.251, *p <* 0.001), AD pathology × global cognition (*r =−*0.545, *p <* 0.001), and M18L × global cognition (*r =* 0.373, *p <* 0.001) were significantly correlated. **b** Regression analyses predicting global cognition as a function of AD pathology in groups of MAP participants having M18L or M18S immunodensities above 90th (green), between 10th and 90th (blue), or below 10th percentile (red). Lines represent best-fit curves and 95 % confident intervals. For M18L, significant correlations between AD pathology and global cognitive function were observed for participants among the lowest (*r =−*0.735, *p <* 0.001, *n =* 31) and intermediate (*r =−*0.512, *p <* 0.001, *n =* 243), but not the highest (*r =−*0.399, *p =* 0.074, *n =* 31), M18L groups. For M18S, statistically significant correlations were seen in all three M18S ranked groups: low (*r =−*0.460, *p =* 0.036, *n =* 31), intermediate (*r =−*0.559, *p <* 0.001, *n =* 245) and high (*r =−*0.509, *p =* 0.021, *n =* 29). **c** Whiskers represent 10th and 90th percentiles of M18L or M18S values, with boxed interquartile ranges crossed by the median, in participants with no- (NCI, *n =* 90), or mild-cognitive impairment (MCI, *n =* 86), or with dementia (DEM, *n =* 132). One-way ANOVA detected a significant effect only for M18L (F_(2305)_ = 8.77, *p <* 0.001). *p < 0.05 and ***p < 0.001, ANOVA followed by Bonferroni’s test. **d** Representative immunoblots of M18L/S and β-actin, with various participants and standard (*std*) samples. Masses are indicated in kDa

We initially conducted regression models (controlling for demographics) to address basic associations between M18 variants, Alzheimer’s disease pathology and global cognitive function. Lower cortical immunodensity of M18L, but not M18S, was associated with the severity of Alzheimer’s disease pathology, and lower cognitive scores (Fig. 4a). Importantly, for those participants above the 90th percentile of M18L the effect of higher levels of Alzheimer’s disease pathology on cognition was not statistically significant, although the number of cases with high pathology was limited, preventing definitive conclusions (Fig. 4b). In contrast, in subjects with M18L score below the 10th percentile, cognitive function was highly sensitive to Alzheimer’s disease pathology. While these observations suggested an interaction between Alzheimer’s disease pathology and M18L cortical levels, the inclusion of a statistical interaction term in the full sample analysis did not render significance. Decay curves for pathology-cognition associations were similar across M18S-ranked MAP participants (Fig. 4b).

Based on prior work showing that select presynaptic proteins were related to cognition in a relatively independent manner [13], we next generated linear regression models to evaluate the impact of M18L on cognitive function, accounting for age-related neuropathology. In these analyses, each of the common age-related neuropathologies was associated to a variable degree with global and/or domain-specific cognitive functions (Table 2, Model 1). Alzheimer’s disease pathology, cerebral macroinfarcts, hippocampal sclerosis, arteriolosclerosis, and atherosclerosis were all associated with poorer cognitive abilities. Lewy bodies and microinfarcts were not, in this subset of MAP participants. Overall, greater M18L cortical immunodensity was associated with better cognitive abilities (Model 2). Cortical amounts of M18L explained an additional 1.7 % of the variance in global cognition, relatively independent of any neuropathology. Of note, the frontal cortical M18L contribution was significant for episodic, semantic and working memory, but not for perceptual speed or visuospatial skills. Variations in M18S immunodensities were not related to global cognition or any of its domains (Model 3); when including both M18L and M18S (Model 4) only the M18L effect remained significant. In a final model (not shown), we analyzed the possible interaction between Alzheimer’s disease pathology and M18L cortical density by adding a statistical interaction term to the model. No significant effect was detected for this interaction term.

**Table 2:** Linear regression models^*a*^ predicting global or single-domain cognitive function

Model no. and terms	Global cognition		Episodic memory		Semantic memory		Working memory		Perceptual speed		Visuospatial ability	
	(*n* = 302)		(*n* = 302)		(*n* = 296)		(*n* = 301)		(*n* = 293)		(*n* = 289)	
	*r* ^2^ or β^*b*^	*p-*value	*r* ^2^ or β	*p-*value	*r* ^2^ or β	*p-*value	*r* ^2^ or β	*p-*value	*r* ^2^ or β	*p-*value	*r* ^2^ or β	*p-*value
Model 1	0.328		0.394		0.208		0.174		0.189		0.121	
AD pathology	−0.872	<0.001	−1.091	<0.001	−0.713	<0.001	−0.634	<0.001	−0.477	<0.001	−0.362	0.014
Macroinfarcts	−0.283	0.016	−0.253	0.039	−0.366	0.011	−0.470	0.001	−0.052	0.681	−0.065	0.657
Lewy bodies	−0.184	0.193	−0.111	0.452	−0.027	0.875	−0.102	0.533	−0.027	0.857	−0.112	0.518
Hipp sclerosis	−0.660	0.001	−1.034	<0.001	−0.662	0.005	−0.143	0.529	−0.687	0.001	−0.315	0.198
Model 2	0.345		0.406		0.226		0.193		0.193		0.123	
AD pathology	−0.793	<0.001	−1.018	<0.001	−0.628	<0.001	−0.549	<0.001	−0.434	0.001	−0.323	0.032
Macroinfarcts	−0.269	0.021	−0.240	0.048	−0.347	0.014	−0.455	0.001	−0.043	0.731	−0.058	0.693
Lewy bodies	−0.141	0.314	−0.071	0.630	0.021	0.900	−0.055	0.735	−0.004	0.981	−0.088	0.611
Hipp sclerosis	−0.658	0.001	−1.032	<0.001	−0.665	0.005	−0.141	0.530	−0.686	0.001	−0.317	0.196
M18L^*c*^	0.0010	0.003	0.0009	0.009	0.0012	0.006	0.0011	0.006	0.0005	0.141	0.0005	0.226
Model 3	0.328		0.392		0.209		0.177		0.188		0.120	
AD pathology	−0.865	<0.001	−1.090	<0.001	−0.707	<0.001	−0.624	<0.001	−0.470	<0.001	−0.357	0.016
Macroinfarcts	−0.290	0.014	−0.255	0.038	−0.372	0.010	−0.483	<0.001	−0.057	0.650	−0.071	0.629
Lewy bodies	−0.175	0.217	−0.108	0.464	−0.011	0.947	−0.086	0.598	−0.018	0.907	−0.100	0.566
Hipp sclerosis	−0.670	0.001	−1.037	<0.001	−0.676	0.005	−0.160	0.480	−0.696	0.001	−0.325	0.184
M18S^*c*^	0.0016	0.331	0.0004	0.794	0.0024	0.247	0.0027	0.150	0.0015	0.389	0.0018	0.383
Model 4	0.343		0.405		0.224		0.191		0.190		0.120	
AD pathology	−0.793	0.001	−1.014	<0.001	−0.629	<0.001	−0.551	<0.001	−0.436	0.001	−0.626	0.021
Macroinfarcts	−0.267	0.022	−0.232	0.057	−0.348	0.014	−0.460	0.001	−0.047	0.714	−0.062	0.673
Lewy bodies	−0.141	0.313	−0.074	0.614	0.022	0.896	−0.053	0.745	−0.002	0.990	−0.085	0.625
Hipp sclerosis	−0.657	0.001	−1.023	<0.001	−0.667	0.005	−0.146	0.515	−0.690	0.001	−0.321	0.189
M18L^*c*^	0.0010	0.006	0.0011	0.006	0.0011	0.013	0.0010	0.017	0.0005	0.217	0.0004	0.343
M18S^*c*^	−0.0003	0.877	−0.0015	0.419	0.0003	0.890	0.0009	0.669	0.0006	0.741	0.0010	0.658

Abbreviations: *AD* Alzheimer’s disease *Hipp*, hippocampal; *M18L/S*, Munc18-1 long/short splice variant

^*a*^All models included terms (not shown) for age, sex, education, microinfarcts, arteriolosclerosis, and atherosclerosis

^*b*^Values are whole model adjusted *r*-squared, or individual β-coefficients for each term

^*c*^Standardized M18L/S values (z-scores) were used

Grouping MAP participants by clinical diagnosis revealed that dementia cases had lower M18L cortical immunodensity (−51 %, *p <* 0.001) than those with no dementia (Fig. 4c–d). Differences were also noted when subjects were graded using either NIA/Reagan or Braak, with lower M18L levels (−77 % and−64 %, respectively) in participants with high probability of Alzheimer’s disease (i.e. subjects falling into NIA/Reagan group 1 and/or Braak stage V–VI) (Additional file 1: Figure S2B). Minimal differences for M18S were observed across clinical diagnoses or postmortem rankings. In further multiple logistic regression analyses we aimed to evaluate the significance of M18L cortical downregulation to the likelihood of clinical dementia (Table 3). As expected, each unit of Alzheimer’s disease pathology, cerebrovascular disease (excluding microinfarcts), Lewy bodies and hippocampal sclerosis increased the odds of dementia (Table 3, Model 1). Each unit of M18L, however, was associated with lower odds of dementia, without altering the effects of neuropathologies (Model 2). The probability of dementia was not related to M18S cortical levels (Model 3), which in turn did not modify M18L effects (Model 4).

**Table 3:** Logistic regression models^*a*^ predicting likelihood of clinical dementia per unit of term

Model terms	Model 1			Model 2			Model 3			Model 4		
	Odds ratio	95 % CI	*p-*value	Odds ratio	95 % CI	*p-*value	Odds ratio	95 % CI	*p-*value	Odds ratio	95 % CI	*p-*value
AD pathology	3.15	1.80–5.73	<0.001	2.84	1.60–5.22	<0.001	3.16	1.81–5.75	<0.001	2.83	1.59–5.21	<0.001
Macroinfarcts	2.16	1.20–3.94	0.010	2.06	1.14–3.79	0.017	2.20	1.22–4.02	0.009	2.05	1.13–3.78	0.018
Microinfarcts	0.73	0.39–1.36	0.326	0.77	0.41–1.43	0.412	0.75	0.40–1.40	0.370	0.77	0.41–1.43	0.409
Arteriolosclerosis	1.30	0.95–1.78	0.098	1.34	0.98–1.84	0.068	1.32	0.97–1.82	0.078	1.33	0.98–1.84	0.071
Atherosclerosis	1.36	1.02–1.82	0.035	1.36	1.02–1.84	0.038	1.34	1.00–1.80	0.046	1.36	1.02–1.84	0.038
Lewy bodies	4.92	1.79–15.40	0.002	4.63	1.67–14.70	0.003	4.93	1.79–15.51	0.002	4.62	1.66–14.66	0.003
Hipp sclerosis	4.39	1.72–12.16	0.002	4.41	1.70–12.39	0.002	4.59	1.78–12.80	0.001	4.38	1.68–12.34	0.002
M18L^*b*^				0.68	0.50–0.90	0.008				0.67	0.47–0.93	0.018
M18S^*b*^							0.84	0.64–1.10	0.208	1.03	0.75–1.43	0.871

Abbreviations: *AD* Alzheimer’s disease, *CI* confidence interval *Hipp*, hippocampal, *M18L/S* Munc18-1 long/short splice variant

^*a*^All models were controlled for age, sex and education

^*b*^Standardized M18L/S values (z-scores) were used

### APP23 mice do not show alterations in M18L/S cortical amounts

**Fig. 5:**
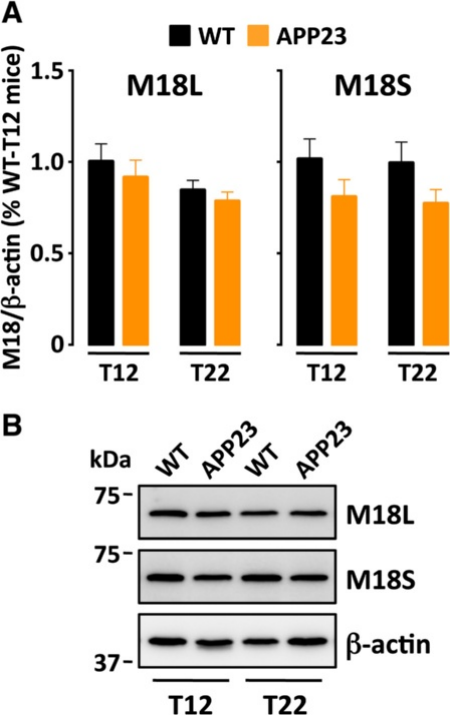
M18 splice variants are not altered in APP23 mice. **a** Immunodensities of M18L/S splice variants were quantified by Western blotting in brain homogenates (frontal cortex) from adult (12-month-old; T12) and aged (22-month-old; T22) wild type (WT) and APP23 transgenic mice. Columns are immunodensity mean values ± standard error (normalized by β-actin) of *n =* 6 mice per group, and represented in percentage to control (T12–WT) animals. Two-way ANOVA only detected a significant effect for genotype (but not age) on M18S (F_(1,20)_ = 4.51, *p =* 0.0462), and a borderline age (but not genotype) effect on M18L (F_(1,20)_ = 3.44, *p =* 0.0784), without specific between-group differences in the following Dunnett’s *post hoc* tests. **b** Representative immunoblots of M18L, M18S and β-actin, with one sample per group. Masses (in kDa) of proximal prestained markers are indicated on the left side of immunoblots

We used APP23 transgenic mice to examine the association between M18L and a common Alzheimer’s disease pathology (i.e. amyloid plaques). These animals develop an Alzheimer’s disease-like syndrome via expression of a mutant APP that causes abnormal, age-dependent extra-synaptic amyloid-β accumulation [48]. M18L/S cortical immunodensities were compared between adult (12-month old) and aged (22-month old) APP23 mice, and age-matched wild type (WT) littermates. Although overt synaptic damage is reported in APP23 mice [48], cortical levels of M18L/S were not significantly different from those in WT controls, at either age (Fig. 5). However, M18L was slightly reduced in aged rats regardless of genotype (−13 to −16 %, *p >* 0.05), while APP23 mice tended to display lower M18S cortical immunodensities regardless of age (−21 to−22 %, *p >* 0.05). These marginal effects were considerably smaller than the M18L loss (−64 %) observed in the DLPFC of MAP participants graded according to Braak’s V–VI stages.

## Discussion

In the present study, variations in frontal cortical M18 levels were associated with cognitive function and the likelihood of dementia in the elderly, beyond the effects of common age-related neuropathologies. Specific loss of M18L (but not M18S), which was found preferentially at GABAergic presynaptic terminals, may initiate synaptic dysfunction and consequent synaptic pathology.

We first characterized biochemical, cellular and anatomical aspects of M18 splice variants. Compared to M18S, M18L showed minimal binding affinity for syntaxin-1 (and hence for the SNARE complex) in two functional assays, co-IP and BN-PAGE. A previous study showed similar, but less pronounced, variant-specific binding affinities to syntaxin-1 [32]. Furthermore, in a yeast two-hybrid setting, depletion of any segment of the M18S (M18L was not reported) amino acid sequence (including the 25 C-terminal residues) completely abolished M18-syntaxin-1 association [49]. It is possible that the present assay conditions (e.g. absence of Ca^2+^) may induce a SNARE conformation that favors M18S relative to M18L binding *in vitro*. Since binding to syntaxin-1 is the major mechanism by which M18 exerts its functions [23, 28, 29], future studies should address this apparent difference in M18 variants in more detail. Given its globular, hydrophilic nature, M18 attachment to cell membranes is thought to occur through syntaxin-1 interaction [50]. Despite the lower affinity for syntaxin-1, M18L seemed confined to the highly hydrophobic, lipid raft-enriched fraction. While the role of these cholesterol-enriched microdomains in regulated exocytosis is unclear, some studies proposed that specific pools of target SNARE proteins may concentrate at lipid rafts, which in turn could discriminate between different kinds of neurotransmitter-containing vesicles [51]. Of note, the M18L C-terminal sequence does not display specific motifs for palmitoylation (cysteine enriched sequences), myristoylation (typically occurring at a N-terminal glycine), or prenylation (typically in a C-terminal cysteine), major post-translational modifications determining membrane localization for globular proteins. Possible explanations for M18L localization at lipid rafts could include a preferential M18L–syntaxin-1 interaction in the biochemical environment of these membrane microdomains, or alternatively, M18L may display greater affinity for another raft-attached protein. Munc18-interacting proteins (Mint1/2) may be key partners in this process, as they bear two membrane-attaching PDZ domains in addition to a M18-binding sequence [52]. Remarkably, Mint1/2 can regulate M18-syntaxin-1 interaction and were found also altered in Alzheimer's diseased postmortem brains [46]. Additionally, the presence of a putative phosphoserine site for Ca^2+^-calmodulin kinase II (CaMKII) activity in the C-terminal tail of M18L, but not M18S, may indicate a differential regulatory mechanism for the M18 variants [39]. This CaMKII activity was presumed to be responsible for the enhanced depression of synchronous vesicle release in M18 null pyramidal neurons rescued with M18L, compared to those carrying M18S only.

Immunohistochemical assays were consistent with tissue fractionation experiments. In contrast to the wide distribution of M18S across subcellular compartments, M18L was restricted to nerve endings and enriched in perisomatic areas. The M18S subcellular and histological distribution was similar to that previously reported for M18 [32, 53], and no cell-type specificity was presumed. The distribution of M18L was consistent with the localization expected for GABAergic presynaptic proteins [54]. Indeed, M18L closely matched VGAT distribution in all brain areas analyzed, especially in the hippocampus, where it was abundantly expressed in the perisomatic area of pyramidal cells. Interestingly, a previous *in situ* hybridization showed greatest expression of M18L in cells within the granule and pyramidal layers of mouse hippocampus [35]. Combined, these experiments indicate that M18L protein is mainly present in the presynaptic terminals of GABAergic interneurons within these hippocampal layers, although we cannot exclude some residual M18L expression in glutamatergic or other neuromodulatory terminals. This finding suggests a qualitative difference between the neurosecretory mechanisms of excitatory and inhibitory neurons. Several epileptic syndromes have been associated with firing abnormalities of dentate gyrus granule cells, likely due to an inappropriate inhibitory tone [55, 56]. This model is consistent with the mutations in M18 related to Ohtahara syndrome [41, 45], a disease accompanied by seizures and severe intellectual disability.

We found marked reductions of M18L, but not M18S, in DLPFC of MAP participants displaying clinical dementia and/or a large burden of Alzheimer’s disease pathology. Pairwise correlations between M18L levels, the burden of Alzheimer’s disease pathology, and cognitive function scores were all significant. Accordingly, participants having high M18L cortical density displayed greater cognitive resistance against the accumulation of Alzheimer’s disease pathology, which may directly implicate M18L in the molecular mechanisms of cognitive reserve. Statistical modeling indicated that loss of M18L contributed moderately to a higher likelihood of dementia and to cognitive impairment. Additionally, including synaptophysin as a covariate did not alter the results, suggesting that loss of M18L is an early event in synaptic pathology. The importance of maintaining appropriate splicing balance between M18L/S isoforms is probably best represented by the dramatic endophenotypes observed in murine strains either lacking or overexpressing the M18L variant [34, 35]. While specific regulatory splicing mechanisms of the *STXBP1* transcript are unclear, other splicing deficits have been associated with aging related neuropathologies and dementia [57].

The predominant expression of M18L in GABAergic terminals suggests that synaptic deficits may be initiated in inhibitory, rather than excitatory synapses. This could be related to the surprising relative upregulation of glutamatergic terminals observed in MCI [58]. Indeed, there is compelling evidence involving GABAergic system in early stages of dementia [59, 60]. By contrast, previous observations in transgenic mouse models suggested that cholinergic and glutamatergic terminals may collapse first as a result of APP-mediated synaptic damage [61]. In the present study, APP23 mice did not display reduced levels of M18L/S, which agrees with previous findings [46]. Thus, cortical loss of M18L may be an independent event from amyloid-β accumulation, and perhaps other common age-related neuropathologies, and could represent a distinct contribution to cognitive impairment, possibly through erosion of cognitive reserve.

## Conclusions

The present study identified the M18L isoform as a relatively specific component of the GABAergic presynaptic machinery. In contrast to the alternatively spliced M18S, which is highly abundant and ubiquitously expressed in inhibitory and excitatory neurons, M18L appears restricted to lipid raft microdomains within the presynaptic compartment, and may have a function independent from its interaction with syntaxin-1. Importantly, M18L, but not M18S, is associated with cognitive function in the elderly, and may contribute to the mechanisms of cognitive reserve that protect against dementia related to age-associated pathologies.

## Methods

### Participants, cognitive evaluations and neuropathological assessments

MAP recruits volunteers without known dementia, living in Chicago (IL, USA) [7]. Since 1997, this study enrolled over 1750 community-dwelling participants. At enrollment, all participants signed an informed consent and an Anatomic Gift Act for organ donation upon death. All protocols were approved by the Institutional Review Board of Rush University Medical Center. The overall follow-up rate is 95 % and the autopsy rate exceeds 80 %. Samples obtained in a consecutive series of autopsies of *n =* 308 participants were included in the present study. A summary of demographic, cognitive and pathological characteristics is listed in Table 1.

Methodological approaches to systematic cognitive, clinical and neuropathological evaluations were extensively reported [7, 62]. Annual cognitive evaluations comprised a battery of 21 standard tests, 19 of which are used to summarize one of the following domains: episodic memory, semantic memory, working memory, perceptual speed or visuospatial ability. For the present work, last valid cognitive tests were used in all analyses. The 19 tests could also be combined into a single variable, based on an average z-score to summarize global cognitive function nearest to death [7, 63]. The final clinical diagnoses of dementia followed the National Institute of Neurological and Communicative Disorders and Stroke and the Alzheimer’s Disease and Related Disorders Association criteria [64], and were made by a board-certified neurologist blind to all pathological data.

Neuropathological examinations documented Alzheimer’s disease-related pathology (neuritic and diffuse plaques, and neurofibrillary tangles), vascular diseases (macroscopic and microscopic infarcts, arteriolosclerosis and atherosclerosis), Lewy bodies, and hippocampal sclerosis [7, 62]. A board-certified neuropathologist made all diagnoses blind to all clinical data. A composite measure of global Alzheimer’s disease pathology was created using a standardized average of neuritic and diffuse plaques and neurofibrillary tangles [62]. The burden of Alzheimer’s disease pathology was also categorized using Braak, CERAD and NIA/Reagan scales.

### Animals

Approval from the UBC’s Animal Care Committee was obtained prior to experiments involving laboratory animals. Adult Sprague–Dawley rats were supplied by Charles-River (Montreal, QC, Canada). Novartis Pharma (Basel, Switzerland) provided APP23 transgenic mice, overexpressing a variant of human APP carrying the ‘Swedish double mutation’ KM670/671NL [48]. Pentobarbital-anaesthetized adult rats, as well as 12- or 22-month old APP23 mice and wild-type (WT) littermates, were killed by decapitation. Hemispheres were separated, and used for electrophoretic or immunohistochemical assays.

### Purification of synaptosomes

Cortical synaptosomes were obtained following standard procedures (see cartoon in Fig. 1d), similar to those originally developed by Grey and Whittaker in the 1960’s [65]. All sucrose solutions described below were HEPES-buffered (4 mM, pH 7.4), supplemented with 1 % of a protease inhibitor cocktail (Sigma, St. Louis, MO, USA), and pre-chilled at 4 °C. Approximately 1 g of human inferior temporal cortex was homogenized in 10 ml of 0.32 M sucrose buffer, using a motorized 20-ml Potter-Elvehjem tissue grinder, with a clearance of 0.13–0.18 mm between the Teflon pestle and the glass chamber. The following centrifugal separations were all performed at 4 °C in an Avanti J-30I high performance centrifuge (Beckman Coulter, Fullerton, CA, USA), equipped with a JA-30.50 fixed angle rotor. Tissue debris, along with cell nuclei, were removed at 1000 × *g* for 1 min (P1). Supernatants (S1) were centrifuged at 17,000 × *g* for 15 min, and the resulting pellets (P2) washed in 0.32 M sucrose and re-centrifuged. P2 fractions were resuspended in 0.32 M sucrose and layered onto a sucrose discontinuous gradient, with 1.2 (bottom), 0.8 M (middle), and 0.32 M (topping) layers. After a 12-h centrifugation at 96,000 × *g*, the interfaces between 0.8 and 1.2 M (IF1, containing the crude synaptosomal fraction), and 0.32–0.8 M (IF2, mainly myelin-coated fragments) were collected. IF1 was again overlaid on 0.32 M sucrose buffer and centrifuged at 30,000 × *g* for 20 min. The resulting pellet, containing purified synaptosomes, was resuspended in 1 ml of homogenization buffer supplemented with 0.5 % TritonX. In all steps, aliquots of each fraction were separated for further SDS-PAGE and immunoblotting analyses.

### Extraction of lipid rafts

Purification of lipid raft-enriched fraction was based on the TritonX-insoluble, SDS-soluble property of these membrane microdomains. The present method, initially designed for separating cytosolic from insoluble, raft-associated α-synuclein [66], offers a simple and quick assay to yield a fraction (S4) of TritonX-insoluble proteins highly enriched in lipid rafts through sequential centrifugation steps [67]. Figure 1f in the main text illustrates the procedure. Briefly, ~150 mg of cortical tissue were homogenized in 1.5 mL of ice-cold 10 mM Tris-buffer, pH 6.8, containing 1 % of a protease inhibitor cocktail (Sigma). Total homogenate (TH) was centrifuged at 31,000 × *g* for 1 h at 4 °C. Supernatant was collected (S1), and the pellet (P1) was resuspended in 1 ml of the same ice-cold Tris-buffered solution supplemented with 0.5 % TritonX. To achieve a homogenous solution, P1 was gently sonicated (F60 Sonic Dismembrator, Fisher Scientific, Waltham, MA, USA). The same centrifugation step was performed and the supernatant (S2) was kept. The pellet (P2) was again resuspended and sonicated in Tris-buffer containing 2 % TritonX, and the centrifugation step was repeated. The subsequent supernatant (S3) was separated and the pellet (P3) homogenized as above in ice-cold Tris-buffer containing 0.5 % SDS. P3 was incubated at 15 °C with rotation for 10 min before a final centrifugation at 31,000 × *g* and at 12 °C was performed for 1 h. The supernatant (S4), enriched in lipid-raft proteins, was collected, and the final pellet (P4) resuspended as above. All fractions were stored at−80 °C until resolved by SDS-PAGE and immunoblotting.

### Antibodies

A list of primary antibodies used appears in Additional file 2: Table S1. Commercial antibodies selectively targeting M18L and M18S variants were raised against their respective C-terminal amino acidic sequences (Fig. 1a), which share 100 % homology across human and rodent orthologues. The isoform-selectivity of these antibodies was previously reported [36], and further characterized by co-immunoprecipitation. Production and characterization of mouse monoclonal antibodies against syntaxin-1, SNAP-25, VAMP and synaptophysin was described elsewhere [68, 69]. Peroxidase- and Alexa-Fluor 488/555/647-conjugated secondary antibodies were from Jackson ImmunoResearch Laboratories (West Grove, PA, USA) or Molecular Probes (Eugene, OR, USA), respectively.

### Immunoprecipitation

Target proteins were immunoprecipitated using protein G-coated magnetic Dynabeads (Life Technologies, Carlsbad, CA, USA), as reported [38]. In each reaction, 50 μg of beads were incubated in phosphate-buffered saline (PBS), supplemented with 0.1 % TritonX, and containing 0.33 μg of anti-mouse IgG (negative control), or antibodies against either M18L or M18S splice variants. Nonspecific binding sites were blocked in the same buffer supplemented with 3 % bovine serum albumin (BSA). In parallel, human cortical samples were ground homogenized in PBS containing 1 % TritonX, and 1 % of protease inhibitors (Sigma), and solubilized for 1 h at 4 °C. Prior to IP reactions, solubilized brain proteins were pre-cleared with antibody-free beads. Antibody-conjugated magnetic beads were combined with excess (2 mg) of pre-cleared brain proteins, and incubated overnight at 4 °C. After washing, IP products were eluted in 25 μl of 20 mM tricine, pH 2.7 to preserve native protein structures and interactions.

### Quantitative immunoblotting

Grey matter samples from the middle-frontal gyrus (Brodmann’s area 46/9) of the DLPFC were obtained at autopsies of MAP participants, following a standard atlas [70]. The DLPFC was selected for its central role in complex cognitive tasks and contribution to age-related cognitive decline [71]. Samples (40–80 mg) were ground-homogenized using a Teflon pestle in ice-cold PBS, pH 7.4, and stored at−80 °C until use [13]. Prior to immunoassays, protein concentrations were determined by DC assay (Bio-Rad, Hercules, CA, USA), and samples adjusted to equal concentrations with homogenization buffer.

Quantification and characterization of M18L/S variants and β-actin in MAP cortical samples was achieved by SDS-PAGE, using 10 % or 12 % minigels (Bio-Rad), followed by immunoblotting, as previously reported [38]. Quantification of other presynaptic proteins was previously reported [13]. Total brain homogenates (and also subcellular fractions or IP products) were combined with equal volumes of 2× loading buffer (100 mM Tris, pH 6.8, 4 % SDS, 0.2 % bromophenol blue, 20 % glycerol, 200 mM β-mercaptoethanol). All samples were boiled for 5 min prior to electrophoretical separations. Preliminary analyses determined that 10-μg protein aliquots of total brain homogenates from DLPFC were optimal for densitometric quantifications [36]. The standard sample (pool of *n =* 132 MAP participants) was spaced-loaded in triplicate in all gels to control for correct loading and protein transfer to the membranes (see Fig. 4d), and also to account for between-gel variability. For quality control, immunoblots were rejected when the coefficient of variation (CV) of in-gel standard samples exceeded 10 %, although typical CV < 5 % values were obtained. For the analysis of brain fractions, equivolumetric amounts were loaded onto the SDS-gels, while 5 μl of IP products were found optimal. All brain samples were resolved in 10–12 % polyacrylamide minigels (Bio-Rad, Hercules, CA, USA). After electrophoresis, proteins were transferred to polyvinylidene difluoride (PVDF) membranes, and subsequently blocked (1 h), and incubated with primary (overnight, 4 °C; see Additional file 2: Table S1) and secondary (1 h; 1:5000) antibodies, in TBS containing 5 % milk and 0.1 % Tween-20. Chemiluminescence was enhanced with commercial reagents (Perkin Elmer, Waltham, MA, USA), and images were digitized using a LAS-3000 Image Reader (Fujifilm, Tokyo, Japan).

In quantitative studies, membrane stripping and reprobing with anti-β-actin antibody was done for housekeeping and data normalization purposes. Densitometric analyses of the immunoblots were done with ImageGauge software, version 4.22 (Fujifilm). Every gel was run with 14 brain samples (including 11 MAP participants randomly selected, and the triplicate standard sample), and a molecular weight ladder (Bio-Rad). Gels containing the same subset of samples were assessed at least twice in different days. For quality control, a minimal between-gel Pearson’s correlation coefficient of *r* = 0.80 was required for M18L/S and β-actin immunoreactivity values. Gels not meeting this criterion were discarded and repeated. For each sample, immunoreactivity of M18L/S (in arbitrary optical density units) was first divided by that of β-actin within the same gel, and then calculated as a percentage of in-gel standards. Mean value between the two different, valid gels was used as a final estimate. This procedures were reported to reduce the experimental variability in studies that used immunoblotting as a quantitative technique for large cohorts of samples [36, 38].

### BN-PAGE

Originally developed to study mitochondrial membrane complexes [72], separation of solubilized brain proteins by BN-PAGE was recently shown suitable to identify and quantify presynaptic complexes in human postmortem tissue [38]. Brain samples were combined with equal volumes of ice-cold 100 mM Bis-tris, pH 7.0, solubilization buffer, containing 50 mM NaCl, 2 mM EDTA, 4 mM 6-aminohexanoic acid, 1 % TritonX, and 1 % protease inhibitor cocktail, and incubated for 1 h at 4 °C with gentle rotation. After centrifugation (16,000 × *g*, 30 min, 4 °C), supernatants or IP products were combined with equal volumes of a loading buffer (0.5 % TritonX, 0.25 % Coomassie brilliant blue G-250, 10 % glycerol). Samples were loaded in 4–16 % gradient NativePAGE precast gels (Novex, Carlsbad, CA, USA). Electrophoresis was run under constant voltage (150 V), at 4 °C, with pre-chilled anode (50 mM Bis-tris, pH 7.0) and cathode (15 mM Bis-tris, 50 mM tricine, pH 7.0, 0.02 % Coomassie dye) buffers. Before transferring proteins and complexes to PVDF membranes, BN-gels were incubated an ice-cold 12 mM Tris, 96 mM glycine buffer, pH 8.3, containing 0.1 % SDS. To remove excess of Coomassie dye, membranes were rinsed in 100 % methanol, followed by immediate rehydration in TBS for 15 min. Following immunoblotting procedures were as described for SDS-PAGE. Molecular markers (NativeMARK, Novex, range 20–1240 kDa) were loaded in all gels to estimate protein complex sizes.

### Immunohistochemistry and immunofluorescence

Forty-μm floating coronal sections (−3.30 to−4.20 relative to bregma) were obtained from paraformaldehyde-fixed rat brain hemispheres [73]. Other floating coronal sections were from the mid-hippocampus of selected cases with or without dementia [74]. Retrieval of the M18L epitope required 20-min incubation at 95 °C in 20 mM Tris buffer (pH 9.0), containing 1 mM EDTA and 0.05 % Tween-20, possibly because of the major localization in TritonX-insoluble subcellular compartments (see Results). Other antigen retrievals were done in 20 mM citrate buffer (pH 6.0) at 80 °C for 15 min. For procedures involving 3,3′–diaminobenzidine staining, we used commercially available kits (Vector Labs, Burlingame, CA, USA), with incubations as reported [75]. Images were acquired with an Olympus BX61 microscope (Olympus, Tokyo, Japan). Immunofluorescence assays were performed as described [74, 76]. A series of orthogonal images were obtained using a LSM 5 Pascal confocal microscope (Zeiss; Jena, Germany). Co-staining of M18L/S isoforms with VGAT and VGLUT1 was assessed to determine presence in inhibitory or excitatory presynaptic terminals, respectively. Appropriate negative controls were included in all experiments.

### Statistical analyses

In quantitative immunoblotting assays, M18L/S immunodensities were first normalized to corresponding β-actin values, and calculated as a percentage of in-gel standards [36, 38]. Linear models were constructed with log-transformed (M18L) and/or standardized (M18L and M18S) values (z-scores). Data transformations did not alter the observed differences between groups.

Multivariate analyses were performed to survey putative associations between M18 isoforms and cognitive, pathological, neurochemical and/or putative confounding variables. An additional exploratory analysis was performed by ranking participants according to their M18L/S cortical immunodensities and comparing pathology-cognition decay curves across subjects with high (above the 90th percentile) and low (below 10th percentile) M18L/S values. Given the potential effect of cortical M18L levels on cognitive performance, we generated multiple linear regression models with cognitive measures as outcomes and pathological and neurochemical variables as predictors. Additionally, using the same predictors, we built logistic regression models with clinical diagnosis of dementia as the outcome. Linear and logistic models were controlled for sex, age and education. Differences in M18 immunodensities between clinically diagnosed or pathologically graded groups were assessed by ANOVA followed by Bonferroni’s *post hoc* test for standardized data, or Kruskal-Wallis followed by Dunn’s *post hoc* tests for non-transformed values. Experiments involving APP23 mice were analyzed with two-way ANOVA, with genotype and age as independent factors, followed by Dunnet’s *post hoc* test.

For colocalization analyses of confocal imaging, we used an ImageJ 2.0 (NIH, Bethesda, MA, USA) built-in method [76, 77]. Comparisons of M18L/S colocalizations across inhibitory *versus* excitatory compartments were performed with paired *t*-tests.

All tests were two-tailed, and *p*-values < 0.05 were considered significant. Datasets were analyzed and plotted with JMP 10.0.2 (SAS Institute, Cary, NC, USA), and/or GraphPad Prism 6.0 (GraphPad Software, La Jolla, CA, USA).

## Additional files

Additional file 1: Figure S1. Immunohistochemical characterization of Munc18-1 splice variants in human dentate gyrus reveals similar cellular and subcellular distributions of M18L and M18S than those in rat brain. Confocal images show a preferential localization of M18L to inhibitory presynaptic terminals, as its immunofluorescence fully overlaps with that of VGAT, but not VGLUT1. M18S shows ubiquitous distribution. **Figure S2.** M18L, but not M18S, immunodensity is reduced in the DLPFC of MAP participants with clinical dementia, compared to those with no or mild cognitive impairment, as well as in those presenting high burden of Alzheimer’s disease pathology, using either NIA/Reagan or Braak scales. (PDF 986 kb) Additional file 2: Table S1. List of both commercially available and locally produced primary antibodies used in the present study. References cited in Table S1 are listed below the table. (PDF 107 kb)

## Abbreviations

ANOVA: Analysis of variance
ANCOVA: Analysis of covariance
APP: Amyloid precursor protein
BN-PAGE: Blue native-PAGE
BSA: Bovine serum albumin
CA: Ammon’s horn
CERAD: Consortium to Establish a Registry for Alzheimer’s Disease
CV: Coefficient of variation
DC: Detergent compatible
DG: Dentate gyrus
DLPFC: Dorsolateral prefrontal cortex
ECL: Enhanced chemiluminescence
EDTA: Ethylenediaminetetraacetic acid
ELISA: Enzyme-linked immunoadsorbant assay
GABA: Gamma-aminobutyric acid
GCL: Granule cell layer
IP: Immunoprecipitation
M18: Munc18-1
M18L/S: M18 long/short splice variant
MAP: Memory and aging project
MCI: Mild cognitive impairment
NCI: No cognitive impairment
PAGE: Polyacrylamide gel electrophoresis
PBS: Phosphate-buffered saline
PMI: Postmortem interval
PPIs: Protein-protein interactions
PVDF: Polyvinylidene difluoride
SDS: Sodium dodecyl sulfate
SNAP-25: Synaptosome-associated protein of 25 kDa
SNARE: soluble *N*-ethylmaleimide-sensitive factor attachment protein receptor
STXBP1: Syntaxin-binding protein-1
TBS: TRIS-buffered saline
TritonX: Triton X-100
VAMP: Vesicle-associated membrane protein
VGAT: Vesicle GABA transporter
VGLUT1: Vesicle glutamate transporter-1
WT: Wild-type
