# Supplementary material for: Loss of Munc18-1 long splice variant in GABAergic terminals is associated with cognitive decline and increased risk of dementia in a community sample
Source: Mol Neurodegener. 2015 Dec 2;10:65. doi: 10.1186/s13024-015-0061-4 (PMC4667524; doi:10.1186/s13024-015-0061-4)
Supplement: Additional file 1: Figure S1. — Immunohistochemical characterization of Munc18-1 splice variants in human dentate gyrus reveals similar cellular and subcellular distributions of M18L and M18S than those in rat brain. Confocal images show a preferential localization of M18L to inhibitory presynaptic terminals, as its immunofluorescence fully overlaps with that of VGAT, but not VGLUT1. M18S shows ubiquitous distribution. Figure S2. M18L, but not M18S, immunodensity is reduced in the DLPFC of MAP participants with clinical dementia, compared to those with no or mild cognitive impairment, as well as in those presenting high burden of Alzheimer’s disease pathology, using either NIA/Reagan or Braak scales. (PDF 986 kb) [file 13024_2015_61_MOESM1_ESM.pdf]

Additional file 1

Additional Figure Legends

Figure S1 Immunohistochemical characterization of M18L and M18S splice variants in human dentate gyrus. All hippocampal sections were from a 91-year old female with confirmed Alzheimer's disease, randomly picked from the limited collection of free floating sections available. (**A**) Representative confocal photomicrographs showing widespread overlapping between syntaxin-1 (STX1) and M18S, and to a lesser extent, M18L. (**B**–**C**) Inhibitory and excitatory presynaptic terminals were respectively co-immunolabeled with antibodies against vesicular GABA (VGAT) and glutamate (VGLUT1) transporters, along with (**B**) anti-M18L or (**C**) anti-M18S antibodies. As shown in the main text for rat brain, in human hippocampal sections M18L was predominantly localized at inhibitory, rather than excitatory synapses. In contrast, M18S displayed ubiquitous distribution across VGAT and VGLUT1 positive terminals. (**A**–**C**) Colors were arbitrarily assigned to maximize overlap visualization. Abbreviations: hil, hilus; sg; stratum granulosum; sm, stratum moleculare. Scale bars: 30 μm.

Figure S2 Reduced M18L, but not M18S, in participants with dementia and/or high Alzheimer's disease (AD) pathology. (**A**–**B**) Densities of M18L and M18S were assessed by quantitative immunoblotting in cortical homogenates (middle-frontal gyrus of the dorsolateral prefrontal cortex) from MAP participants, and values were further normalized by β-actin. (**A**) Scatterplot depicting the semi-logarithmic association between M18 variants. Dots represent β-actin-normalized immunodensity values of M18L (X-axis) by M18S (Y-axis) for each MAP participant. Individual clinical diagnosis of no- (NCI, n = 90; green), or mild-cognitive impairment (MCI, n = 86; blue), or dementia (DEM, n = 132; orange) are indicated; (**B**)

Participants were grouped either by (left) clinical diagnosis (NCI/MCI/DEM); (middle) NIA/Reagan (stages 4–3: $n = 124$; stage 2: $n = 135$; stage 1: $n = 49$); or (right) Braak (stages 0–II: $n = 57$; stages III–IV: $n = 179$; stages V–VI: $n = 72$). Some neuropathological stages according to NIA/Reagan or Braak were clustered to balance groups (see actual sizes for each corresponding stage in Table 1). Dots are individual values, and crossing lines represent mean values ± standard errors for each group of subjects. The Kruskal-Wallis test detected significant effects for M18L in each of the three classifications (clinical diagnosis: KW-statistic = 19.1, $p < 0.001$; NIA/Reagan: KW-statistic = 24.6, $p < 0.001$; Braak: KW-statistic = 16.2, $p < 0.001$), whereas for M18S a marginal effect was observed only when participants were graded by NIA/Reagan (KW-statistic = 6.7, $p = 0.034$). *$p < 0.05$, **$p < 0.01$ and ***$p < 0.001$, Kruskal-Wallis followed by Dunn's *post hoc* test.

Figure S1

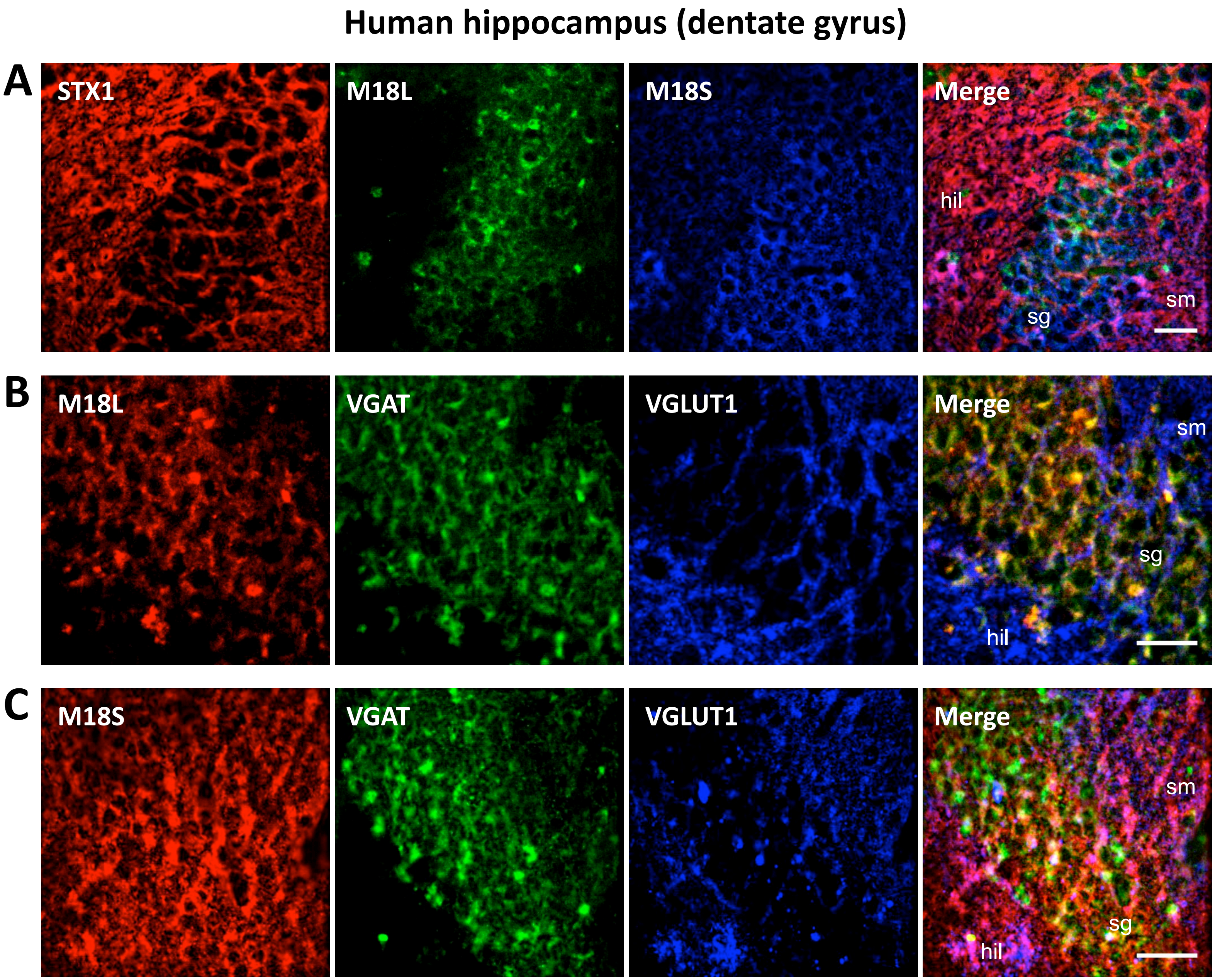
Human hippocampus (dentate gyrus)
A
STX1
M18L
M18S
Merge
hil
sg
sm
B
M18L
VGAT
VGLUT1
Merge
sm
sg
hil
C
M18S
VGAT
VGLUT1
Merge
sm
sg
hil

Figure S2

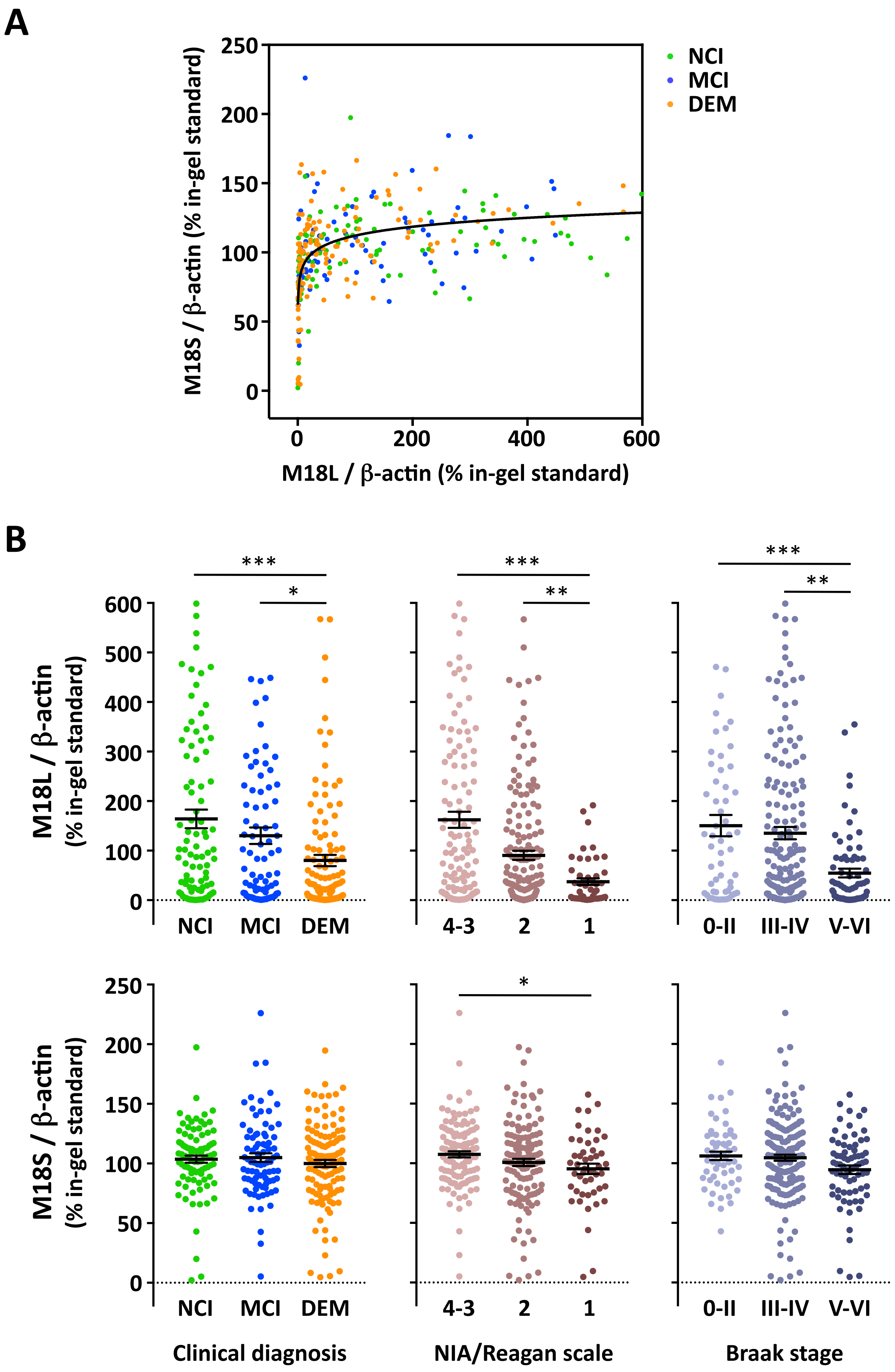
A
M18S / β-actin (% in-gel standard)
250
200
150
100
50
0
0
200
400
600
M18L / β-actin (% in-gel standard)
NCI
MCI
DEM
B
M18L / β-actin
(% in-gel standard)
600
500
400
300
200
100
0
***
*
***
**
***
**
NCI
MCI
DEM
4-3
2
1
0-II
III-IV
V-VI
M18S / β-actin
(% in-gel standard)
250
200
150
100
50
0
*
NCI
MCI
DEM
4-3
2
1
0-II
III-IV
V-VI
Clinical diagnosis
NIA/Reagan scale
Braak stage
