# Supplementary material for: Loss of Munc18-1 long splice variant in GABAergic terminals is associated with cognitive decline and increased risk of dementia in a community sample
Source: Mol Neurodegener. 2015 Dec 2;10:65. doi: 10.1186/s13024-015-0061-4 (PMC4667524; doi:10.1186/s13024-015-0061-4)
Supplement: Additional file 2: Table S1. — List of both commercially available and locally produced primary antibodies used in the present study. References cited in Table S1 are listed below the table. (PDF 107 kb) [file 13024_2015_61_MOESM2_ESM.pdf]

Additional file 2

Table S1. Commercial and locally produced primary antibodies used in the present study.

Commercially available antibodies

Target protein	*Immunogen*	*Host*	*Clone*	*Subclass*	*WB dilution*	*IHC dilution*	*Catalogue no*	*Vendor*
M18L	Residues 591–603 of human M18L	Goat	Polyclonal	–	1:2,000	1:500	PAB6504	Abnova
M18S	Residues 577–594 of human M18S	Rabbit	Polyclonal	–	1:4,000	1:1,000	M 2694	Sigma
FosB	Internal residues mapping mouse FosB	Rabbit	Polyclonal	–	1:1,000	–	sc-48	Santa Cruz
NeuN	Mouse brain nuclear extract	Mouse	A60	IgG1	1:1,000	1:500	MAB377	Chemicon
MBP	Residues 70–89 of human myelin basic protein	Mouse	SMI-94	IgG1	1:2,000	–	SMI-94R	Covance
α-synuclein	Residues 15–123 of rat synuclein-1	Mouse	42/α-Syn	IgG1	1:1,000	–	610787	BD Biosciences
VGAT	Residues 75–87 of rat VGAT	Rabbit	Polyclonal	–	–	1:500	131 002	Synaptic Systems
VGAT	Residues 2–115 of rat VGAT	GPig	Polyclonal	–	–	1:500	131 004	Synaptic Systems
VGLUT1	Residues 456–560 of rat VGLUT1	Rabbit	Polyclonal	–	–	1:500	135 302	Synaptic Systems
VGLUT1	Residues 456–560 of rat VGLUT1	Mouse	317D5	IgG2a	–	1:500	135 311	Synaptic Systems
β-actin	Residues 2-16 of human β-actin	Mouse	AC-15	IgG1	1:10,000	–	A1978	Sigma

Locally produced mouse monoclonal antibodies

Target protein	*Immunogen*	*Host*	*Clone*	*Subclass*	*WB dilution*[a]	*IHC dilution*	*Reference*
Syntaxin-1	Crude human brain immunoprecipitate	Mouse	SP7	IgG2a	1:100	3 µg/ml	[1]
Syntaxin-1	Crude human brain immunoprecipitate	Mouse	SP6	IgG1	1:100	–	[1]
SNAP-25	Crude human brain immunoprecipitate	Mouse	SP12	IgG1	1:100	–	[1]
VAMP	Crude human brain immunoprecipitate	Mouse	SP10	IgM	1:10	–	[1]
Synaptophysin	Schizophrenia human brain homogenate	Mouse	EP10	IgG1	1:100	–	[2]

Abbreviations: GPig, Guinea pig; M18L/S, Munc18-1 long/short splice variant; MBP: myelin basic protein; NeuN, neuronal nuclear protein; SNAP-25, synaptosome-associated protein of 25 kDa; VAMP, vesicle-associated membrane protein; VGAT, vesicular GABA transporter; VGLUT1, vesicular glutamate transporter-1

[a]Dilutions are from hybridoma cell culture supernatants.

References

1. Honer WG, Hu L, Davies P: **Human synaptic proteins with a heterogeneous distribution in cerebellum and visual cortex.** *Brain Res* 1993, **609**:9–20.

2. Honer WG, Kaufmann CA, Kleinman JE, Casanova MF, Davies P: **Monoclonal antibodies to study the brain in schizophrenia.** *Brain Res* 1989, **500**:379–383.
